# Commentary: Preparation, characterization and application of rod-like chitin nanocrystal by using p-toluenesulfonic acid/choline chloride deep eutectic solvent as a hydrolytic media

**DOI:** 10.3389/fbioe.2020.00505

**Published:** 2020-06-12

**Authors:** Wen-Hao Jiang, Wei-Ming Gu, Mei-Jie Xiong, Kang-Ping He, Xiao-Ying Xu, Wen-Xi Zhang, Rui-Feng Shen, Lin-Hao Lai, Yong-Si Lv, Shi-Lin Cao

**Affiliations:** ^1^School of Food Science and Engineering, Foshan University, Foshan, China; ^2^Sustainable Biochemical and Biosynthetic Engineering Center, Foshan Wu-Yuan Biotechnology Co., Ltd., Guangdong Biomedical Industrial Base, Foshan, China

**Keywords:** enzyme immobilization, chitin nanocrystal, deep eutectic solvent, lipase, biocatalysis

## Introduction

Chitin nanocrystal (ChiNC) is a unique bio-based nanomaterial extracted from chitin. This nano-biopolymer is biocompatible, biodegradable, and antimicrobial. In recent decades, ChiNC exhibits great potential in several fields, such as bio-medicine (Sahraee et al., 2017) and biocatalysis (Huang et al., 2018). In the recent publication from our research group, a green preparation method for ChiNC was established by using deep eutectic solvent (DES) as media and the relative ChiNC product was proved to be an excellent enzyme carrier for lipase immobilization. In this commentary, we would like to add some information for the design of the recyclable ChiNC nanocrystal enzyme carriers by using DES as media, which provides insight in enzyme immobilization.

## Challenges in Developing Mild and Green Methods in Preparing CHINC Enzyme Carriers

According to the previous studies, acid hydrolysis (Gopalan and Dufresne, 2003; Goodrich and Winter, 2007) and oxidation (Fan et al., 2008) are common ways of producing ChiNC. However, there are some drawbacks in these methods.

In the acid hydrolysis, abundant acid medias (mineral acids and/or organic acids) were used. Unfortunately, these acid medias were difficult to recycle. This leads to waste of resources and pollution. For example, during a typical acid hydrolysis process of ChiNC, treating 1 g of chitin materials would consume about 30 mL 3 mol/L HCl at first. In the following process, about 5–10 times of water (150 to 300 mL) was required for dilution (thus, the HCl media cannot be reused). In addition, this process needs to be repeated for more than three times. Moreover, 3.6 g of NaOH was used to neutralize the HCl media. In general, to treat 1 kg chitin, about 30 L 3M HCl solution, 3.6 kg solid NaOH, and more that 600 L water was consumed. Besides, as for TEMPO(2,2,6,6-Tetramethyl-1-piperidinyloxy)-oxidation, to fabricate the ChiNC with abundant carboxyl groups, 1 mol TEMPO and more than 100 L water are needed to treat 1 kg of chitin. Thus, designing the ChiNC preparation process by using hydrolytic media with low volatility and reusability is urgently needed to solve this problem.

Deep eutectic solvents (DESs) are mixtures consisting of hydrogen bond donor and acceptor pair with low volatility. Thus, DESs exhibit great potential as recyclable reaction medias. Moreover, it is important to choose a good diluent. The following requirements need to be met as a suitable diluent: (1) the diluent should be completely miscible with DES to form a homogeneous mixture solution; (2) since the high viscosity of the DES resulted in lower sedimentation efficiency, the diluent should have relatively low viscosity to reduce the viscosity of the mixture media; (3) in order to separate and recycle the dilute and DESs from the mixture media, the dilute should be volatile with a suitable boiling point. In our previous experiments, ethanol was found to fulfill all the above requirements. Also, it is noteworthy that the ChiNC is easy to sedimentate in the ethanol media.

**Figure 1:**
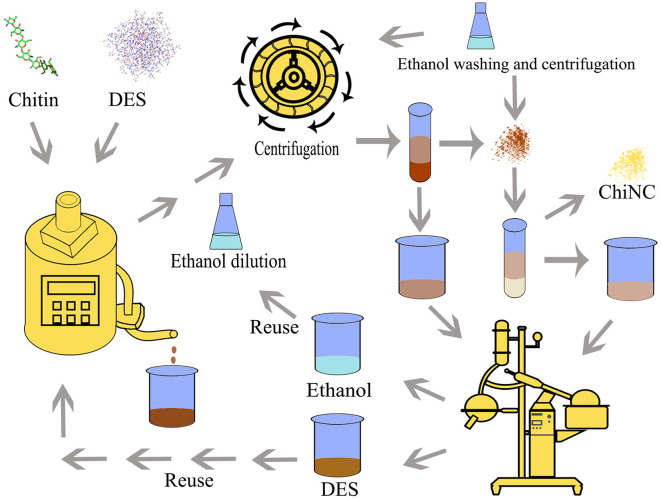
The scheme of the ChiNC preparation process by using DES as media.

Thus, in our latest publication, a type of acidic DESs (p-toluenesulfonic acid/choline chloride, PTA**-**ChCl) was used. The preparation process was designed as follows (Figure 1): (1) chitin raw material was treated with PTA**-**ChCl DES to form ChiNC; (2) the mixture was diluted by about absolute ethanol and centrifuged to separate the ChiNC product and mixture media (containing PTA-ChCl and absolute ethanol); (3) the ChiNC product was furtherly washed with absolute ethanol; (4) PTA-ChCl and absolute ethanol was separated from the mixture media obtained in step (2) by distillation or rotational evaporation. In these preparation process, the PTA-ChCl and ethanol can be reused.

## Future Perspective

There are several aspects that need further exploration in using DESs to prepare ChiNC nanocarriers. Firstly, the effects of different DESs treatments on the physical and chemical properties of the ChiNC nanocarriers need to be further investigated. This can help researchers to design more efficient and greener ChiNC preparation processes. Secondly, a clear understanding of the interaction between ChiNC enzyme carriers and enzyme is lacking. Until now, the mechanism of the interaction of the enzyme and carriers at the atomic and molecular level still require further investigation. Thus, molecular dynamic simulation and quantum chemistry is a promising way to better explain the underlying mechanism at the atomic and/or molecular level.

## Author Contributions

S-LC, W-MG, W-HJ, M-JX, K-PH, X-YX, W-XZ, R-FS, Y-SL, and L-HL wrote the manuscript. S-LC and W-MG edited the manuscript. W-MG manufactured the Figure.

## Conflict of Interest

S-LC was part-time employed by the company Foshan Wu-Yuan Biotechnology Co., Ltd. The remaining authors declare that the research was conducted in the absence of any commercial or financial relationships that could be construed as a potential conflict of interest.
